# Postbiotics Production of Candidate-Probiotic *Lactiplantibacillus plantarum* AC131 with Renewable Bio Resources

**DOI:** 10.3390/life13102006

**Published:** 2023-10-02

**Authors:** Svetla Danova, Dragomir Yankov, Lili Dobreva, Ana Dobreva, Nadya Armenova, Apostol Apostolov, Milka Mileva

**Affiliations:** 1 Institute of Microbiology “Stephan Angeloff” Bulgarian Academy of Sciences, 1113 Sofia, Bulgaria; stdanova@yahoo.com (S.D.); ldobreva@microbio.bas.bg (L.D.); 2 Institute of Chemical Engineering, Bulgarian Academy of Sciences, 1113 Sofia, Bulgaria; yanpe@bas.bg (D.Y.); n.armenova@gmail.com (N.A.); a_apostolov@iche.bas.bg (A.A.); 3 Institute for Roses and Aromatic Plants, Agriculture Academy, 6100 Kazanlak, Bulgaria; anadobreva@abv.bg

**Keywords:** dried distillers’ grains with soluble (DDGS), *Lactiplantibacillus plantarum*, postbiotics, lactic acid, wastewaters from rose oil production

## Abstract

*Lactiplantibacillus plantarum* is a versatile specie, well known as a producer of lactic acid (LA) and other metabolites with biotechnological significance. The present work characterizes growth and lactic acid production of the candidate-probiotic strain *L. plantarum* AC131, from Bulgarian white brined cheeses. Different nutritional media with ingredients from renewable resources—reduced sugars from dried distillers’ grains with soluble (DDGS) and waste waters from the water-vapor distillation of Bulgarian *Rosa alba* L. and *Rosa damascena* Mill. essential oil—were assessed. The results obtained showed significant LA production (up to 95% conversion) in modified MRS broth with reducing sugars from DDGS hydrolysates. The addition of *R. alba* L. and *R. damascena* Mill. distillation effluents stimulated the growth and biological activity of postbiotics produced by *L. plantarum* AC131. In both experimental approaches, a statistically significant inhibition (from 20 to 60%) of *E. coli* HB 101 growth was found during 24 h exposure and a variable effect on the biofilm formed. In conclusion, reducing sugars from DDGS hydrolysates can be successfully used as a carbon source for lactic acid production. In the case of fermentation without pH control, the process is product inhibited, while with pH control, nearly full conversion was achieved. Postbiotics produced during the process of fermentation showed a variety of biological activity and inhibitory effects on the growth of *Escherichia coli* HB 101.

## 1. Introduction

Lactic acid bacteria (LAB) are widely accepted as probiotics (pro-for and bio-life). According to the FAO/WHO definition, (2014) they are “Live microorganisms, which when administered in adequate amounts, confer a health benefit on the host” [1]. They contribute to the functional activity of key organs and systems in the human body, through gut homeostasis [2]. Due to their role in the modulation of the immune response, probiotics represent an effective alternative in the treatment of inflammatory gastrointestinal diseases [3]. On the other hand, changes in European Community legislation pose the necessity of strict scientific evidence for the advertised health effects of foods and supplements containing probiotics, as well as a better understanding of the pathways and mechanisms through which beneficial bacteria influence health. 

Attention to the favorable role of probiotics beyond their viability (or so-called parabiotics—dead cells/cells fragments or compounds) has been paid only in the last several years [4]. In the case of probiotics, a new innovative objective are postbiotics. According to the last ISAPP definition, postbiotics are “inanimate microorganisms or bacterial cell components” and that post-fermentation by-products such as lactic acid and short-chain fatty acids (SCFA) positively affect the host. The beneficial effects of secreted metabolites during the fermentation by live bacteria or released cell-derived fragments/molecules after microbial lysis, as factors promoting quality of life, well-being, health, and longevity have attracted interest too [5]. Several metabolites such as lactic acid (LA), other volatile organic acids, bioactive peptides, or soluble low-molecular mass antimicrobials possess physiological benefits to the host, in parallel with a high biotechnological importance. The antimicrobial activity of LAB strains due to the produced variety of postbiotics is a promising base for food safety. With special attention to the LAB with a broad spectrum of activity, different strains with dairy and/or human origin were characterized [6,7]. The dominance of *Lactiplantibacillus plantarum* in autochthonous microbiota of traditional Bulgarian dairy products has been pointed out [8]. During fermentation, they produce a variety of metabolites, thus contributing to the preservation and safety of the food. *L. plantarum* is among one of the most common species in various habitats from vegetables and dairy products, through feed and food to the human body, as a natural inhabitant, in the vagina, in saliva, and in the gastrointestinal tract of people. Such high ecological adaptability is based on their high genomic plasticity. This species is unique with evolutionary adopted “life island” genes, coding metabolic activity. Thus, in different conditions, a large range of carbohydrates can be metabolized and as a result, lactic acid and other post metabolites can be obtained. Due to these post metabolites, the species has been proven to improve the immunity of the host and, in addition to maintaining the balance of the gastrointestinal flora, it stimulates the effective absorption of nutrients, relieves lactose intolerance, and contributes to cholesterol lowering [3,4]. 

Several effector molecules of food-fermenting LAB, from various habitats, are thought to be a basis of probiotic potential, but this link has not been conclusively established. They may be produced in different food matrices or in vivo, in human GIT. The LA produced lowers the pH in the gut, contributing to the initial barrier mechanisms limiting the development of pathogens. Strains that quickly acidify the environment in their capacity as starters and or bioprotective adjuncts for the lactic-acid fermentation of fruits and vegetables, silages, and other perishable products were sought. This and other functional characteristics are the basis of the selection of probiotics for the protection of the uro-vaginal tract. LAB producers of lactic acids and SCFAs based on renewable sources are among the innovative biotechnological challenges. Moreover, 2-Hydroxypropionic (lactic) acid is a versatile chemical with diverse applications in many industries as a building block or probiotic. 

Many modern industries generate waste products containing valuable ingredients for subsequent microbial processes. For example, dried distillers’ grains with soluble (DDGS) are the main by-product in bioethanol production. Annually, more than 70 millions of tones DDGS are produced worldwide [9]. Various lignocellulosic wastes have also been used as a substrate for LA production—sugarcane bagasse [10], wheat straw [11], orange peel wastes [12], and spent coffee grounds [13]. Nagarajan, et al. [14] have explored the possibility of using acid-thermal seaweed hydrolysates for LA production. The efficiency of the bioconversion of hydrolysates was in the order red microalgae > green microalgae > brown macroalgae with a degree of conversion > 0.8 g/g. Recently, Yankov [9] has discussed different aspects of LA production—microorganisms, substrates, process organization, genetic engineering, etc. 

On a global scale, Bulgaria is famous as one of the main producers of rose oil. The most popular technological method, which produces oil with unique qualities, valuable for perfumery and cosmetics, is water-steam distillation. The yield of essential oil is low—from 40 kg of rose flower, 1 g of rose oil is obtained—and as a waste product of this scheme there is the separation of bulky waste, i.e., spent rose petals as a solid residue, as well as wastewater (liquid residue) [15]. Unfortunately, they often end up in sewers and drainage systems and become an environmental hazard as pollutants. However, in recent years, opportunities for the utilization of this waste have been sought because a significant content of biologically active ingredients has been found in it. Rose wastewater (RWW) contains significant amounts of water-soluble phytocompounds that could serve as a good, natural, inexpensive source of biologically active compounds and high-value products [16]. 

In the present work, a way was sought to utilize two waste products for the purposes of fermentation processes and LAB’s post metabolites production. With this aim, initially several Bulgarian LAB strains isolated from different habitats were studied and *L. plantarum* AC131 was selected. To the best of our knowledge, the effect of such additives on the LA production and activity of this candidate probiotic strain was not investigated.

The goal of this work was to characterize the growth and LA production of the candidate-probiotic strain *L. plantarum* AC131. Different nutritional media with ingredients from renewable sources—reduced sugars from DDGS and effluents from the water-steam distillation of essential rose oil— were assessed as bioactive compounds. The antagonistic effects of the produced new metabolites on the growth and biofilms of *Escherichia coli* inhibition were assessed in vitro. 

Due to the understanding of the role of the protective biofilms formed by the LAB, the possibility of components of the modified renewable products to stimulate biofilms was evaluated. In this regard, we included in the work the evaluation of the role.

## 2. Materials and Methods

### 2.1. Dried Distillers’ Grains with Soluble (DDGS) as a Source of Reducing Sugars

DDGS possesses a broad spectrum of compounds with impact for microbial nutrients—lignocellulose—36%, protein—23%, starch—5%, fibers—9%. In this study, DDGS from wheat from the distillery Almagest AG, Bulgaria, was used as a raw material. Spent grains were pretreated with 1% H_2_SO_4_ (1:5 solid to liquid ratio) in an autoclave at 121 °C for 30 min. The liquid phase was separated by centrifugation and after neutralization and adequate dilution it was used for culture media preparation. Usually, acid pretreatment results in a solution with a reducing sugars content of about 60 g/L. The mean composition of reducing sugars was as follows: glucose—3%; xylose, galactose, and mannose—15%; arabinose—26%; disaccharides—12%; and oligosaccharides—44% (Yankov, D. unpublished data). The acid hydrolysates were used in experiments for studying lactic acid production. With the purpose to increase the content of monosaccharides, the enzyme hydrolysis step was added after acid hydrolysis. Two cellulases (Hostazym X/Huvepharma/and Alphamalt TTC/Muhlenchemie/) were used at their optimum conditions. Enzyme hydrolysates were used in the experiments for biological activity assessment. 

The concentrations of reducing sugars in the DDGS hydrolysates were measured according to a modified Bertrand’s method described previously [17]. 

### 2.2. Preparation of Wastewaters from the Industrial Cycle of Water-Steam Distillation of Rose Oil

The wastewater was collected after the distillation of rose flowers, as described previously [18]. Briefly, the semi-industrial installation of the Institute for Roses and Aromatic Plants at Kazanlak City, Bulgaria, was used. The process parameters were as follows: raw material 8–10 kg; hydro module 1:4; flow rate 16–20 mL/min; duration 150 min. The wastewaters were sealed and stored in a cool place until the next stage of the investigation.

### 2.3. Microorganisms, Culture Media, and Growth Conditions

The *Lactobacillus* strain AC131 from the collection of the Laboratory “Lactic Acid bacteria & Probiotics”—The Stephan Angeloff Institute of Microbiology, Sofia, Bulgaria, was studied. The strain AC131 was isolated from a home-made sample of white brined cheese from the village of Arda, Bulgaria [6]. *L. plantarum* AC131 was stored in De Man Rogosa Sharp (MRS, Hi Media, India) broth supplemented with glycerol 20% *v*/*v* at −20 °C. Prior to experiments, the strain was pre-cultured twice in MRS broth (Merck, Darmstadt, Germany) (pH 6.2 at 37 °C). In preliminary tests, these conditions were found to be optimal. Different media based of the modification of MRS were designed for post metabolite’s production study (Table 1). The mMRS var. 1 (variant 1) was used as a base for all modifications, using different carbon sources. In all experiments with newly modified media, a control commercial MRS broth (Merck) was applied.

Lactose and reducing sugars from DDGS hydrolysates were used as carbon sources in the variants of the modified MRS broth (Table 1). The wastewater from rose oil production was added 10% *v*/*v* to the mMRS var. 1 broth.

The *Lactobacillus* and *Escherichia coli* HB101 (K2) growth at 37 °C (Nuve EN 400 incubator) for 24/48 or 50 h was monitored spectrophotometrically in 96-well microplates for initial media assessment using microplate reader INNO, CheongJu, Korea, at 600 nm.

### 2.4. Fermentation Processes and Lactic Acid Production

#### 2.4.1. LA Production

Experiments for lactic acid production without pH control were carried out in 300 mL Erlenmeyer flasks with 100 mL medium, inoculated with a 10% 24 h culture grown in inoculum LA broth, with lactose as a carbon source (Table 1). Fermentations with pH control were carried out in the bioreactor Bioflo, New Brunswick Scientific (Edison, NJ, USA), with a working volume of 300 mL at static conditions at 37 °C.

#### 2.4.2. Determination of Biomass Concentration

The biomass concentration of the samples was determined by measuring the absorbance at 620 nm. The spectrophotometer SPEKOL 11 (CARL ZEISE, Yena, Germany) was used for this purpose. The concentration was calculated from a calibration curve, made after measuring the absorbance of a suspension of constant weight dried biomass, with a precisely defined concentration (mg/mL).

#### 2.4.3. HPLC Analysis

High-performance liquid chromatography was used to determine the reducing sugars and lactic acid concentrations. The samples were analyzed on a chromatographic system consisting of a Smartline S-100 pump, a refractometric detector—Perkin-Elmer LC-25RI, and specialized EuroChrom software v. 3.05. The analyses were performed in isocratic mode at 70 °C. The columns used were an Aminex HPX-87H (for lactic acid), and an Aminex HPX-87C (for reducing sugars), both from Bio-Rad, 300 × 7.8 mm. A 0.005 M solution of H_2_SO_4_ (in the case of the HPX-87H column) and deionized water (in the case of the HPX-87C column), were used as the mobile phase at a flow rate of 0.6 mL/min. Before injection, samples were centrifuged and filtered through a 0.22 μm filter.

#### 2.4.4. In vitro Assessment of Antimicrobial Activity 

The agar wall diffusion method in appropriate protocols for different pathogens was applied, under modification according to Dobreva et al. [19]. In vitro tests were carried out in triplicate with the following samples (i) non-neutralized, acid cell-free supernatants (CFS) from a late exponential phase in MRS broth (marked as a CFS) and (ii) in modified MRS (mMRS var. 3) with reducing sugars from DDGS; (iii) whey fractions (WF) from commercial cow milk (1.5% fat), fermented 18 h with 5% (*v*/*v*) overnight Lactobacillus milk culture and (iv) parabiotic–thermally destroyed (autoclaved at 110 °C–15 min at 0.8 atm pressure) living cells from stationary phase culture in MRS broth. The inhibitory activity against the indicator microbes *Staphylococcus aureus* WDCM 00032; *Escherichia coli* HB101; *Pseudomonas aeruginosa* PAO 1; and *Salmonella enterica* subsp. *enterica* serovar Typhimurium WDCM 00031 Vitroids™ (*S. Typhimurium*) was calculated as described by Maulidayanti and Mubarik [20]. The inhibitory activity, based on the measured sterile zone around the wall, was expressed in AU (activity unit). One AU/mL of tested samples of post metabolites was calculated using the equation:

AU/mL = Lz − Ls/V, where Lz = surface of the clear zone (mm^2^); Ls = area of well (mm^2^); V = volume of samples (mL).

Different growth media were used for the test pathogens, as follows: brain heart infusion (Difco) broth and agar for *Salmonella* Typhimurium and *Staphylococcus aureus*; tryptic soya broth (HiMedia, Mumbai, India) and agar for *Ps. aeruginosa*; and MacKonkey agar (Merck, Darmstadt, Germany) for *Escherichia coli*. All test cultures were stored at −20 °C in cultural media supplemented with 20% *v*/*v* glycerol. Prior to the experiments, the overnight fresh cultures at 37 °C were obtained and were standardized to 0.5 McFarland to achieve ~10^6^–10^7^ CFU/mL inoculum in the agar plates.

#### 2.4.5. LAB Biofilm—Formation

The biofilm formation in mMRS broth supplemented with reducing sugars (var. 3) or rose wastewaters (10% *v*/*v*) was monitored in a 96-well microplate (Orange Scientific, Braine-l’Alleud, Belgium) with 200 µL appropriate media (according to the test-microorganisms), containing 1 × 10^6^ CFU/mL, with the Crystal Violet test (CV) as described by Georgiev et al. [21].

## 3. Results and Discussion 

*L. plantarum* is one of the species with reach a metabolic potential often reported as probiotic [22]. The beneficial characteristics of several *L. plantarum* strains, from the laboratory collection of The Stephan Angeloff Institute of Microbiology, have been characterized in vitro. They were isolated from traditional Bulgarian fermented products—“katak” [23], and white brined cheese [6,24]. *L. plantarum* AC131, from artisanal Bulgarian white brined cheese, was assessed as a candidate-probiotic with antifungal activity [25] and a high adhesion capability [26]. Different variants of modified MRS broth were designed (Table 1). Two renewable and low-cost resources (DGGS and rose wastewater) were used as appropriate compounds for media modification.

### 3.1. Growth and Lactic Acid Production Using Sugars from DDGS

**Figure 1:**
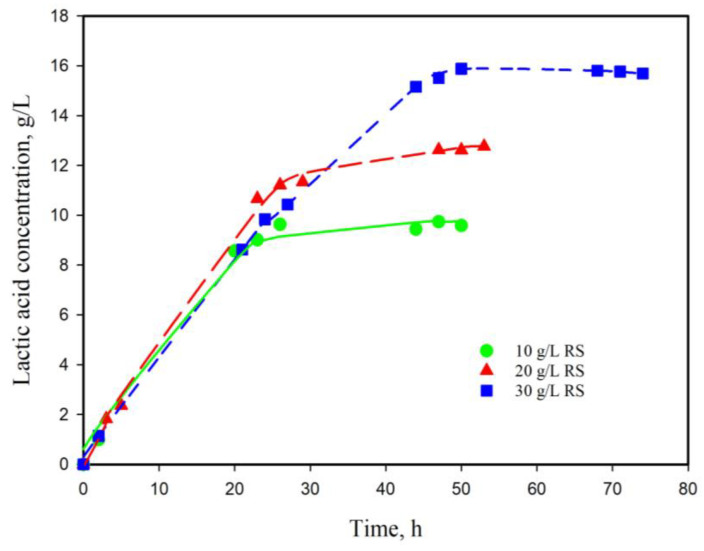
Lactic acid production from different amount of reducing sugars (RS) from DDGS hydrolysates added in mMRS broth var. 3 (Table 1).

In the present investigation, the growth and lactic acid production of *L. plantarum* AC131 was studied. Into the growth medium (mMRS var. 3), reducing sugars from DDGS were added (Table 1). DDGS represents a rich source of fermentable sugars after appropriate pretreatment. The results from the assay for lactic acid production from the obtained hydrolysates with different initial concentrations of reducing sugars are summarized in Figure 1. Experiments were carried out without pH control during the fermentation and the growth level was also monitored.

From the results presented, it is clearly seen that in the absence of pH control, complete conversion is not achieved, probably due to substrate and product inhibition. The increase in initial substrate concentration prolongs the duration of the fermentation. While at a 10 g/L substrate concentration, the maximum in LA produced is reached at about 25 h, in the case of 20 g/L the plateau is reached by 35–37 h and at about 50 h at 30 g/L. The inhibition influences the degree of conversion as well—at 10 g/L, the conversion is about 95%, while it is about 64% at 20 g/L and only 53% at a 30 g/L substrate concentration. The corresponding conversion degrees on mMRS with lactose at the same concentrations (var. 2) were 99, 70, and 55% and the maximum of LA concentrations were achieved a little bit faster—at 22, 30, and 45 h.

**Figure 2:**
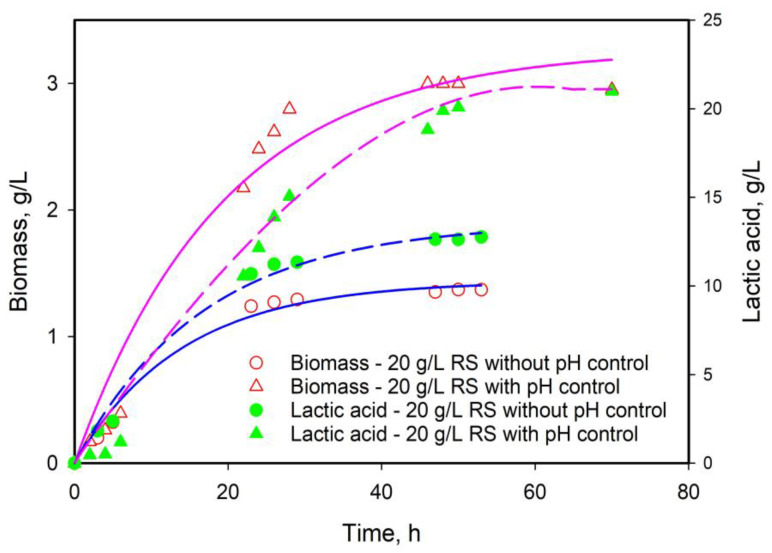
Influence of pH control on lactic acid production—20 g/L RS as substrate. Circles—without pH control, triangles—with pH control, open symbols—biomass, closed symbols—lactic acid.

**Figure 3:**
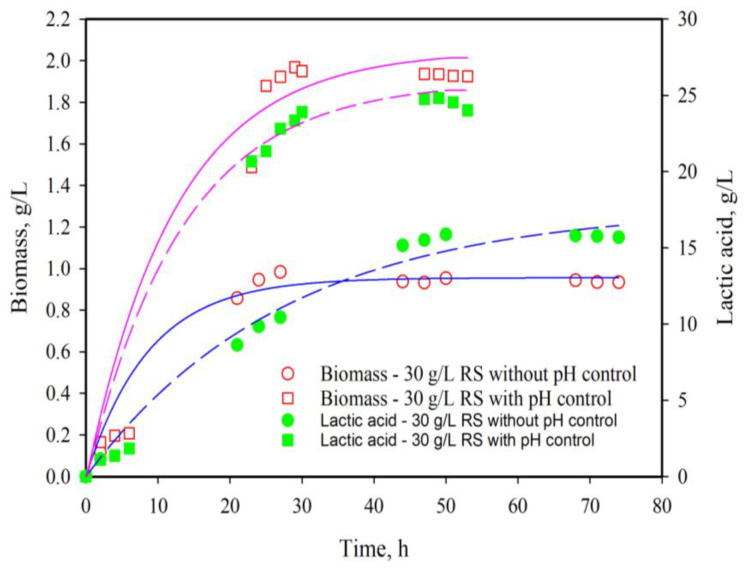
Influence of pH control on the growth and *L. plantarun* AC131 lactic acid production at 30 g/L RS as substrate. Circles—with pH control, rectangles—without pH control, open symbols—biomass, closed symbols—lactic acid.

In order to avoid product inhibition, fermentations with pH control were conducted. The results are presented in Figure 2 and Figure 3. In both cases (20 and 30 g/L substrate concentration), nearly complete conversion was obtained by about 40 h.

The presented results clearly show that the process for lactic acid production from distilleries’ spent grains’ hydrolysates used as a substrate is feasible and further investigations on a pilot plant scale are necessary.

Lactic acid fermentation using different substrates leads to the production of different postbiotics. By modulating the composition of the nutrient matrix, better growth can be achieved in combination with an economically advantageous process. With this aim, we investigated the growth and mode of production of several active post metabolites produced by this versatile LAB species.

In line with the directives of the pressing green economy, opportunities for zero-waste new technology are being sought. Brewer’s spent grain (BSG) and DDGS are among the most abundant agro-industrial by-products and hydrolysates obtained by different pretreatment methods have been used for LA production. Mussatto, et al. [27] used BSG consecutively treated with diluted sulfuric acid, sodium hydroxide, and cellulase as a substrate for fermentation. The authors reported that the supplementation of the hydrolysates with nutrients and control of the pH led to an increase in productivity of up to 35.5 g/L. Pejin, et al. [28] utilized enzyme hydrolysates of dried BSG and investigated the influence of initial reducing sugars and pH control on the LA production. The addition of yeast extract significantly increased the LA yield. The maximum LA production (39.4 g/L) was achieved from 54 g/L RS with 50 g/L yeast extract. Djukić-Vuković, et al. [29] investigated the effect of different fermentation parameters on lactic acid production from liquid distillery stillage by *Lactobacillus rhamnosus* ATCC 7469 and reported a yield of 73.4%. Certain results for LA production from renewable resources are summarized in Table 2.

### 3.2. Growth of L. plantarum in Modified Media Supplemented with a Roses Flowers Distillation Wastewater (RWW)

**Figure 4:**
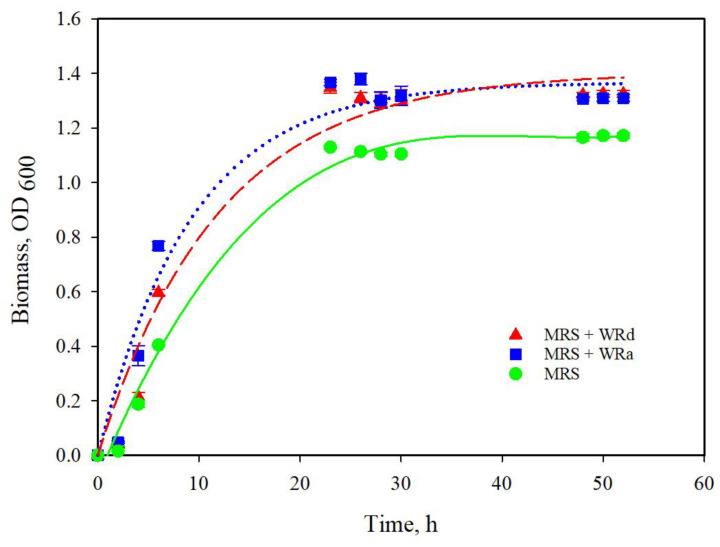
Growth of *L. plantarum* AC131 in MRS (var. 1) broth and mMRS (var. 1) supplemented with wastewater from rose oil production.

Wastewaters from the distillation of *Rosa alba* L. (WRa) and *Rosa damascena* Mill. (WRd) essential oils were used to modify MRS broth (var. 1). The growth (Figure 4) and biological activity of the post metabolites produced during the fermentation were estimated in vitro.

The wastewater from both rose species, added at 10% *v*/*v* to the MRS broth, does not show a significant effect on the growth. However, in our previous studies we found that the wastewater produced after distillation of Bulgarian oil roses *R. damascena* Mill. and *R. alba* L. exhibited valuable biological properties—radical scavenging activity, good antiherpes simplex virus type 1 (HSV-1) activity [18,30]. Moreover, they retain an excellent toxicological safety profile (low cytotoxic effect) against standard non-tumorigenic cell lines such as human HEK-293 (embryonic kidney cells) and mouse cell line (CCL-1 fibroblasts, which are recommended as a standard for cytotoxicity assessment in Annex C of ISO 10993-5). The concentration range of effects found (0.04–0.92% *v*/*v*) is much lower than most of the maximum permissible concentrations for tissue culture cells (0.2–3.4% *v*/*v*). The effluent showed no significant antiproliferative effect against *Staphylococcus aureus* and a low activity against Gram-negative bacteria, with low bactericidal and antifungal effects [30].

### 3.3. In Vitro Assessment of Biological Activity of L. plantarum AC131 Postbiotics Obtained during the Fermentation

In the present study, a broad spectrum of antibacterial activity, including Gram (−) and Gram (+) microorganisms, was observed (Table 2). The highest activity against *Staphylococcus aureus* was determined in modified media with reducing sugars (Table 3).

The presented results showed that different antimicrobials might be produced. Neutralized cell-free supernatants (CFS) showed activity only against *E. coli*, while the acid-filtered spent culture from different growth media was more active. Lactic acid is one of the major metabolites during fermentation. This metabolite is involved in the barrier mechanism against pathogens in vivo and possesses a key role in gut and liver homeostasis [31].

**Figure 5:**
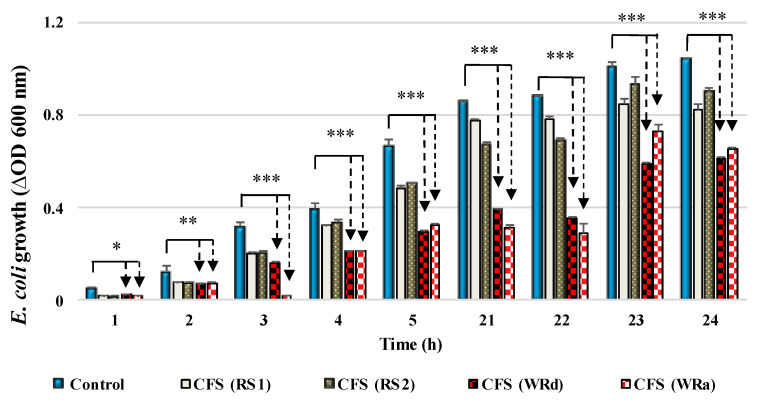
In vitro effect of postbiotics of *L. plantarum* AC131 on the growth of *E. coli* HB101 in BHI supplemented with reducing sugars (RS obtained with acid hydrolysis and two cellulases—RS1 and RS2, respectively) or with wastewaters from *Rosa alba* L. (WRa) or *Rosa damascena* Mill. (WRd). * *p* < 0.05 vs. controls; ** *p* < 0.01 vs. controls; *** *p* < 0.001 vs. controls. As a control, BHI medium (Difco) supplemented with MRS broth (10% *v*/*v*) was used. The arrows show statistical significance of tested samples v/s the controls (Student *t*-Test).

In addition to the high growth and LA production rate noted (Figure 2 and Figure 3), in modified MRS broth (var. 2 with added WWR) and with added reducing sugars from DDGS a significant inhibitory effect on the growth of *E. coli* HB101, must be pointed out (Figure 5). At the same time, in the pure media, fermentation by *L. plantarum* AC131 (mMRS var. 3 with RS and var. 2 m MRS with WWR) did not inhibit *E. coli* growth. Most likely, different active metabolites were produced during the fermentation in modified cultural media with removable bio resources. Further characterization, however, is needed and is still in progress. The stable anti-*E. coli* activity may possess a practical significance, due to the proven safety of WWR. In our previous study, we investigated the cytotoxic/genotoxic and anti-cytotoxic/anti-genotoxic potential of these products in different experimental treatment regimens [32]. The results obtained showed that the genotoxic activity of the distillation effluents of *R. alba* and *R. damascena* was low. Both types of waste products exhibited anti-genotoxic effects against N-methyl-N0-nitro-N-nitrosoguanidine as a direct mutagen, and cytoprotective/genoprotective effects to promote a reduction in DNA damage. As can be seen, these effluents fall into the category of bio-pollutants, but they contain valuable bioactive compounds and exhibit interesting biological properties [32]. We related the exhibited biological activities to the total flavonoid content of the two waste products—tannins in *R. damascena* were 1.61 ± 0.05 mg/mL, and in *R. alba* tannins were 2.16 ± 0.35, flavonoids 1.14 ± 0.01 mg/mL and 1.01 ± 0.01 mg/mL, and total polyphenols 7.2 ± 0.2 and 7.6 ± 0.3 mg/mL, respectively [18]. 

Cell-free supernatants of *L. plantarum* KU200656 (KU200656) from Korean fermented kimchi inhibited growth and biofilm formation by pathogenic bacteria such as *Staphylococcus aureus*, *Listeria monocytogenes*, and *Escherichia coli* [33]. *L. plantarum* isolated from pickles and fermented dairy products has been found to possess antimicrobial and anti-biofilm effect against the multidrug resistant strain of uropathogenic *E. coli* U 12 [34]. In addition to the antibacterial effect, *L. plantarum* broth culture was found to inhibit methicillin-resistant *S. aureus* strain growth and decrease biofilm thickness as evidenced by scanning electron microscopy [35].

**Figure 6:**
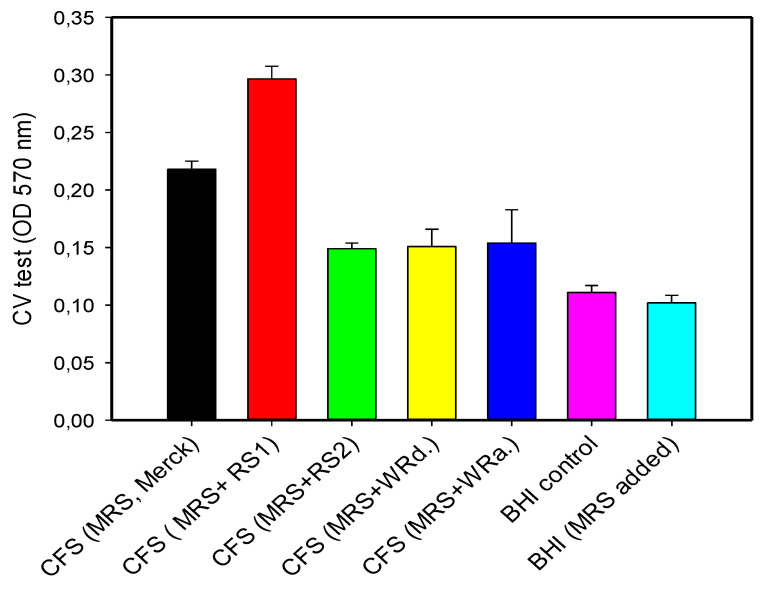
In vitro assessment of *E. coli* HB 101 biofilm formation during the cultivation in BHI supplemented with post metabolites (10% *v*/*v*) of *L. plantarum* AC 131.

In our study, *Lactobacillus’* post metabolites did not inhibit *E. coli* HB101 biofilms formed after 24 h cultivation in BHI broth supplemented with spent cultures—filtered cell-free supernatants (CFS) from *L. plantarum* AC131 fermentation in modified growth media (var. 2 and var. 3 mMRS broth). The biofilm formation of *E. coli* HB101 was assessed using a microplate CV assay after 24 h of cultivation in BHI broth supplemented with 10% *v*/*v* of CFS. The results (Figure 6) showed even slow to significant stimulation in the presence of a high content of RS1, probably due to the resting nutrient for the pathogen.

Biofilm communities are much more resistant to various abiotic influences and antibiotics, as well as host immune defense, and antimicrobial peptides [36]. According to different studies, biofilm formation is strain and species specific [37]. Spreading across surfaces is important for the colonization and establishment of biofilm communities of bacteria that are particularly resistant to antimicrobials [38,39]. 

## 4. Conclusions

Characterization of the growth and postbiotics production of the candidate-probiotics strain *L. plantarum* AC131 using modified MRS broth with renewable bioresources is a promising approach for its further implementation. DDGS hydrolysates represent a perspective source of saccharides as a carbon source for LA production. *L. plantarum* AC131 grows and produces LA with more than 90% conversion on modified media containing sugars from DDGS hydrolysates. The process under pH control ameliorates the degree of conversion. In addition, a higher antimicrobial activity against *E. coli* HB 101 and certain Gram (+) and Gram (−) pathogens was observed. The postbiotics produced in modified media with RS obtained after acid and enzyme (with two cellulases) or with rose wastewater do not inhibit *E. coli* biofilm formation; however, they do stimulate *L. plantarum* AC131 growth. The obtained results are a promising basis for technologically relevant new media using renewable bioresources. However, an additional further scale-up study is also needed.

## Figures and Tables

**Table 1:** Content of media (in g/L) used the growth and production study of LAB.

Components g	Cultural Media—the Control and Modified MRS Variants			
	MRS (Merck)	mMRS Var. 1	mMRS Var. 2	mMRS Var. 3
Peptone	10.0	10.0	10	11
Meat extract	8.0	-	-	-
Yeast extract	4.0	5.0	4.0	4.5
D (+) Glucose	20.0	20.0	-	-
Lactose monohydrate	-	-	11.0	
Reducing sugars	-	-	-	10 -30
K_2_HPO_4_	2.0	2.0	0.25	0.25
KH_2_PO_4_	-	-	0.25	0.25
Tween 80	1.0	-	-	-
Ammonium citrate	2.0	2.0	-	-
CH_3_COONa	5.0	5.0	10	10
MgSO_4_·5H_2_O	0.2	0.2	0.1	0.1
MnSO_4_	0.04	0.04	0.05	0.05
FeSO_4_·7H_2_O		-	0.05	0.05
dH_2_0	1 L	1 L	1 L	1 L
pH (at 25 °C)	6.8	6.5	6.5	6.5

**Table 2:** LA production from renewable resources.

Substrate Origin	Strain	Initial Sugars Concentration, g/L	LA Produced, g/L	Literature
Brown macroalgae	*Lactiplantibaillus plantarum 23*	15	11.65	14
Bagasse	*Lactobaillus delbrueckii*	21	16.5	10
Wheat straw	*Rhyzopus oryzae*	25	16.6	11
Orange peel wastes	*Lactobaillus delbrueckii*	10	8.7	12
Spent coffee grounds	*Sacharomyces cerevisiae*	17.2	11.2	13
Brewer’s spent grains	*L. delbrueckii* UFVH2B20	51	35.5	27
Brewer’s spent grains	*Lacticaseibacillus rhamnosus* ATCC 7469	54	44.4	28
Distillery stillage	*L. rhamnosus* ATCC 7469	25	18.6	29
Dry distillery grains	*L. plantarum* AC131	10 20 30	9.85 19.9 24.5	This work

**Table 3:** Antimicrobial activity (expressed as AU/mL) of post metabolites produced in modified MRS broth and milk against different test pathogens.

Microorganisms	Activity of the Variants Tested (AU/mL)			
	WF	aCFS-MRS	Lysates	MRS var. 3 + RS
*St. aureus*	291.43	1416.92	1134.32	16583.13
*Ps. aeruginosa*		2357.45	551.95	nd

## Data Availability

Not applicable.
